# The Phage Lysin PlySs2 Decolonizes *Streptococcus suis* from Murine Intranasal Mucosa

**DOI:** 10.1371/journal.pone.0169180

**Published:** 2017-01-03

**Authors:** Daniel B. Gilmer, Jonathan E. Schmitz, Mya Thandar, Chad W. Euler, Vincent A. Fischetti

**Affiliations:** Laboratory of Bacterial Pathogenesis and Immunology, The Rockefeller University, 1230 York Avenue, New York, New York, United States of America; Universidade de Lisboa Faculdade de Medicina, PORTUGAL

## Abstract

*Streptococcus suis* infects pigs worldwide and may be zoonotically transmitted to humans with a mortality rate of up to 20%. *S*. *suis* has been shown to develop *in vitro* resistance to the two leading drugs of choice, penicillin and gentamicin. Because of this, we have pursued an alternative therapy to treat these pathogens using bacteriophage lysins. The bacteriophage lysin PlySs2 is derived from an *S*. *suis* phage and displays potent lytic activity against most strains of that species including serotypes 2 and 9. At 64 μg/ml, PlySs2 reduced multiple serotypes of *S*. *suis* by 5 to 6-logs within 1 hour *in vitro* and exhibited a minimum inhibitory concentration (MIC) of 32 μg/ml for a *S*. *suis* serotype 2 strain and 64 μg/ml for a serotype 9 strain. Using a single 0.1-mg dose, the colonizing *S*. *suis* serotype 9 strain was reduced from the murine intranasal mucosa by >4 logs; a 0.1-mg dose of gentamicin reduced *S*. *suis* by <3-logs. A combination of 0.05 mg PlySs2 + 0.05 mg gentamicin reduced *S*. *suis* by >5-logs. While resistance to gentamicin was induced after systematically increasing levels of gentamicin in an *S*. *suis* culture, the same protocol resulted in no observable resistance to PlySs2. Thus, PlySs2 has both broad and high killing activity against multiple serotypes and strains of *S*. *suis*, making it a possible tool in the control and prevention of *S*. *suis* infections in pigs and humans.

## Introduction

The zoonotic Gram-positive pathogen *Streptococcus suis* causes sepsis and meningitis in pigs and humans. *S*. *suis* serotypes 2, 14 and 9 have been most often associated with disease, with serotype 9 causing pig infections primarily in Europe [1–3]. While serotype 2 strains are responsible for over 90% of *S*. *suis* infections in pigs, both serotype 2 and 9 strains have been associated with increasing reports of zoonotic transmission from pigs to humans [4] [5]. Pigs are colonized in the upper respiratory tract with *S*. *suis* (particularly the tonsils and nasal cavities) and may transmit the pathogen to both humans and piglets causing pneumonia, septicemia, or meningitis within days resulting in 20% death if untreated [3, 6, 7]. The earliest human case was reported in 1968; since then, over 700 human cases have been reported in multiple continents with a significant 5–20% mortality rate [4, 8–10]. Humans and pigs can be systemically treated with penicillin or gentamicin with success, but *S*. *suis* isolates resistant to these antibiotics have emerged worldwide [3, 11, 12]. Currently, there is no vaccine for *S*. *suis* [3], and as such, *S*. *suis* is developing a more consistent presence in human populations and are becoming more difficult to treat.

Alternative therapies must be developed to mitigate the sharp increase in antibiotic resistance among Gram-positive bacteria including *S*. *suis*. Novel antimicrobial strategies include enzyme-based antibiotics (“enzybiotics”) such as phage lytic enzymes (endolysins, or simply “lysins”). Bacteriophages encode lysins that hydrolyze the peptidoglycan bonds in the bacterial cell wall after phage progeny replicate inside the infected host bacterium [13]. Disruption of the cell wall leads to hypotonic lysis of the bacteria and release of viral progeny [14]. When applied exogenously, purified lysins are able to access and degrade the bonds in the cell wall of Gram-positive bacteria, because they lack the outer membrane found in Gram-negative organisms [14]. Lysins are quite efficient, killing bacteria faster than any known non-chemical agent [15–17]. With some exceptions (PlySs2 being one), lysins typically demonstrate high specificity, with lethal activity directed against the species that the lysin-encoding phage infects [14–16, 18, 19]. Therefore, lysins should not disrupt the host’s normal flora as would broad spectrum antibiotics [14].

Two phages (Ss1 and SMP) infecting *S*. *suis* have been previously isolated and studied. Harel et al. induced a siphoviral prophage (Ss1) from the genome of a serotype 2 strain 89–999 (the first phage isolated from *S*. *suis*), however, the identity of its lysin remains undetermined [20]. More recently, Ma and Lu isolated a lytic phage (SMP) from nasal swabs of healthy pigs and sequenced its 36 kb genome [21]. SMP, demonstrated a limited host range, infecting only 2/24 *S*. *suis* strains within serotype 2. The same group later PCR-cloned and recombinantly expressed the SMP lysin (LySMP); the enzyme demonstrated bacteriolytic activity *in vitro* against several *S*. *suis* serotypes. Unfortunately, the recombinant LySMP did not fold properly, and was only active in the presence of reducing agents, limiting its potential for *in vivo* use [22]. Since then, it has been tested against biofilms only *in vitro* [23].

Of the currently reported *S*. *suis* lysins, only one described activity against more than 3 serotypes of *S*. *suis (Ply30)* [24], but none showed that the lysins they developed could decolonize animals *in vivo* [20–23]. Recently, our lab discovered a phage lytic enzyme from an *S*. *suis* prophage with broad activity against various pathogenic Gram-positive pathogens, which was named PlySs2 (**p**hage **ly**sin from ***S*. *s****uis* **2**) [25]. It was shown to protect mice from a mixed bacteremic infection of methicillin-resistant *Staphylococcus aureus* (MRSA) and *Streptococcus pyogenes*, neither of which were found to develop resistance to PlySs2 *in vitro* [25]. In this report, we characterize the activity of PlySs2 against *S*. *suis* and test this lysin’s ability to decolonize *S*. *suis* from nasal passages.

## Materials and Methods

### Bacterial strains

All *S*. *suis* strains were stored at -80°C (S1 Table). The strains were grown in brain heart infusion (BHI) broth at 37°C for all tests. Luria-Bertani broth was used to cultivate *Escherichia coli*. All media was from Becton Dickinson, and Company (Sparks, MD).

### Cloning, expression, and purification of PlySs2

The lysin gene *PlySs2* was PCR-cloned from genomic DNA into the pBAD24 expression plasmid, and transformed into *E*. *coli* TOP10 cells (Invitrogen). As detailed by Gilmer et al. [25], the clone was then grown, expressed, and the PlySs2 lysin was purified.

### PlySs2 activity against *S*. *suis* serotypes and strains

Strains of bacteria were grown to log-phase at 37°C and brought to an optical density (OD_600_) of ~1.0 with 15 mM phosphate buffer (PB), pH 8.0 (buffer A) as measured in 96-well microtiter plates (Falcon). From these bacterial stocks, 245 μl were added to each well of a 96-well microtiter plate. In triplicate for every strain, each well received 5 μl of PlySs2 at 1.6 μg/μl (8 μg, resulting in a final concentration of 32 μg/ml). In preliminary experiments, 32 μg/ml provided the best resolution in determining PlySs2 activity. Corresponding triplicate wells received 5 μl of 15 mM PB, pH 6.7 (buffer B) control vehicle. *S*. *suis* 7997 served as a positive control for each trial. At room temperature, a Spectramax Plus 384 (Molecular Devices) took spectrophotometric readings (at λ = 600 nm, i.e., OD_600_) of each well every minute over an hour. The degree of turbidity reduction (OD_600_) in the test wells indicated the amount of lysin activity. To normalize and combine values from multiple tests, the final OD_600_ of the treated samples was divided by the final OD_600_ of the untreated samples. An OD_600_ ratio of 1.0 indicates no lysis, while an OD_600_ ratio of ~0.02 indicates complete lysis.

### Bactericidal assay

Log-phase bacteria were centrifuged and washed 1× in buffer A and adjusted to an OD_600_ of 0.1 (= 0.5 McFarland, ~10^8^ CFU/ml) in buffer A with a SmartSpec™ Plus Spectrophotometer (Bio-Rad). 100 μl aliquots of the cell suspension were distributed in 96-well polypropylene microtiter plate (Costar). PlySs2, at 64 μg/ml, or buffer B control vehicle was added to the wells in triplicate for each strain. Plates were sealed and shaken at 37°C every minute for 1 hour. At this time, 10-fold serial dilutions of each well were plated on BHI agar and incubated at 37°C. Resultant colonies were enumerated after 18 h. The bactericidal effect was calculated as the difference between vehicle-treated and PlySs2-treated CFU for each strain.

### MIC analysis

The protocol of Wiegand, et al. [26] was used with adjustments to determine minimum inhibitory concentrations (MICs). Briefly, each strain was grown in BHI and adjusted to ~5 × 10^5^ cells/ml in BHI and distributed into 4 wells of a 96-well round bottom polystyrene microtiter plate. In each of two wells corresponding to each strain, either sterile-filtered lysin or control vehicle was added [26]. The lysin concentration varied from 0.5–1,024 μg/ml PlySs2. The plates were then incubated for 18 h at 37°C. The MIC was the lowest or minimum concentration of lysin that prevented the formation of a cell pellet (a measure of growth) on the bottom of the wells. The MICs were also colorimetrically confirmed by staining the bacteria in the wells with alamarBlue® vital dye following the manufacturer’s protocol (Invitrogen).

### *In vitro* resistance studies

A published protocol to test the *in vitro* development of antibiotic-resistance was followed [25, 27, 28]. Briefly, *S*. *suis* was grown in the presence of doubling concentrations of PlySs2 over 8 days in BHI broth, and the PlySs2 MIC was tested daily to determine if resistance was acquired. On the first day, bacteria at ~5 × 10^8^ CFU/ml were grown overnight at 37°C in 10 ml BHI containing 1/32× the PlySs2 MIC for the given strain. On the second day, the culture was split into two equal portions. The cells of one aliquot were pelleted and resuspended in 10 ml fresh BHI media with double the concentration of PlySs2 (i.e., 1/16× the PlySs2 MIC on day 2). This aliquot was re-incubated at 37°C overnight. The fluctuation observed in this assay was +/- 1x MIC. Over 8 days, the concentration of PlySs2 was serially doubled from 1/32× the initial MIC (on day 1) to 4× the initial MIC (on day 8) (i.e., the concentrations on each of the 8 days were 1/32×, 1/16×, 1/8×, 1/4×, 1/2×, 1×, 2×, and 4×).

A sample of the second half of the aliquot was spread on BHI agar containing the PlySs2 MIC for that strain. After incubation at 37°C, 4 colonies were selected from the BHI agar plate to determine if a 4-fold increase in PlySs2 MIC was achieved for that strain, indicating the emergence of resistance. The protocol was repeated with gentamicin as an antibiotic resistance control for each *S*. *suis* strain, because *S*. *suis* strains develop resistance to gentamicin *in vitro* [12].

### *In vivo* murine model

A *S*. *suis* nasal mucosal colonization model described by Seitz, et al. [29], was used to test the *in vivo* efficacy of PlySs2 to decolonize *S*. *suis* strain 7997. This strain was made spontaneously resistant to 200 μg/ml streptomycin, through passage of bacteria in media containing increasing concentrations of the antibiotic, to distinguish it from other organisms found in the murine nasal mucosa. Next, 4-week old female CD-1^®^ mice (Charles River, Wilmington, MA) were acclimated for 7 days then given drinking water containing 5 mg/ml streptomycin. After two days, mice were anesthetized and 12.5 μl of 1% acetic acid was delivered to each nostril. An hour later, they were administered 10 μl (~1 × 10^9^ CFU) of mid log-phase (OD_600_ of ~0.5, concentrated ~100x) *S*. *suis* in 50 mM PB, pH 7.4 (buffer C) in each nostril. An aliquot of each inoculation stock was serially diluted and plated to Columbia blood agar plates to confirm the actual bacterial inoculation titer.

Twenty-four hours after *S*. *suis* administration, the animals were randomly divided into 4 treatment groups. To each nostril, we delivered 10 μl of either: buffer C alone (group 1), 5 mg/ml PlySs2 in buffer C (0.1 mg total) (group 2), 5 mg/ml gentamicin in buffer C (0.1 mg total) (group 3), or a combination of 2.5 mg/ml PlySs2 and 2.5 mg/ml gentamicin in buffer C (0.05 mg total of each) (group 4). Twenty-four hours after treatment, all mice were euthanized by CO_2_-inhalation. The nasal passage of each mouse was surgically removed post-mortem, bisected to expose the sinuses, and vortexed in 500 μl of buffer C. Serial dilutions were then streaked on 5% sheep blood plates (containing 200 μg/ml streptomycin) and incubated at 37°C for final colony counts.

#### Animal care

All protocols in this *in vivo* study were approved by The Rockefeller University’s Institutional Animal Care and Use Committee. In this animal model the animals were only nasally colonized with the *S*. *suis* and not expected to become ill, so they were monitored every 8 hours during the procedure and no early endpoint protocols were required. Also, since the animals do not become ill with this model, no methods were necessary to alleviate any distress the animals would have encountered during the experiment. As expected, no animals died or became visually ill during this experiment. The Rockefeller University Laboratory animal facilities are fully accredited by the American Association for Accreditation of Laboratory Animal Care. Animals are maintained in accordance with the applicable portions of the Animal Welfare Act and the DHHS "Guide for the Care and Use of Laboratory Animals. Veterinary care is under the direction of a full time resident veterinarian boarded by the American College of Laboratory Animal Medicine.

## Results

### Broad lytic activity

**Fig 1:**
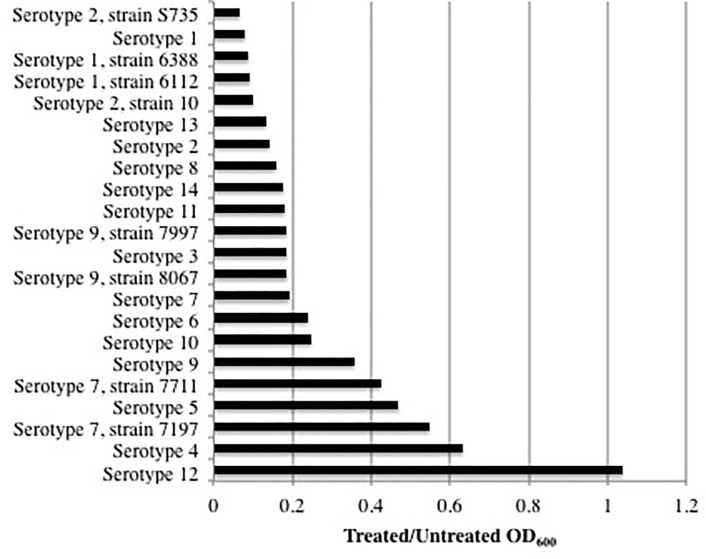
PlySs2 displayed activity against almost all strains of *S*. *suis*. Bacteria in logarithmic growth were exposed to 32 μg/ml PlySs2 for 30 minutes in PB (for 60-minute readings, see S3 Fig). The activity was measured by OD_600_ reduction. To normalize and combine values from multiple tests, the final OD_600_ of the treated samples was divided by the final OD_600_ of the untreated samples. An OD_600_ ratio of 1.0 indicates no lysis, while an OD_600_ ratio of ~0.02 indicates complete lysis.

Purified PlySs2 was tested against 22 strains representing 8 serotypes of *S*. *suis* to assess its range of lytic activity. Over 30 minutes, 14 of 22 strains were reduced to an OD_600_ ratio of ≤0.2 from a starting OD_600_ of ~1.0 (Fig 1). Readings taken after 60 minutes showed the same relative reduction in OD_600_ (S1 Fig). This group of PlySs2-sensitive *S*. *suis* strains included the type strain S735, and the pathogenic serotype 2 and 9 strains. The reduction in OD_600_ ratio of other *S*. *suis* strains was between 0.2 and ~0.6. Serotype 12 was the only strain that exhibited a negligible decrease in optical density. Both serotype 2 and 9 strains S735 and 7997 respectively, revealed a significant drop in OD_600_ at >4 μg/ml PlySs2 when tested in a time-dependent lytic assay at various PlySs2 dosages (S2 and S3 Figs).

### Efficacy of PlySs2 against *S*. *suis*

**Fig 2:**
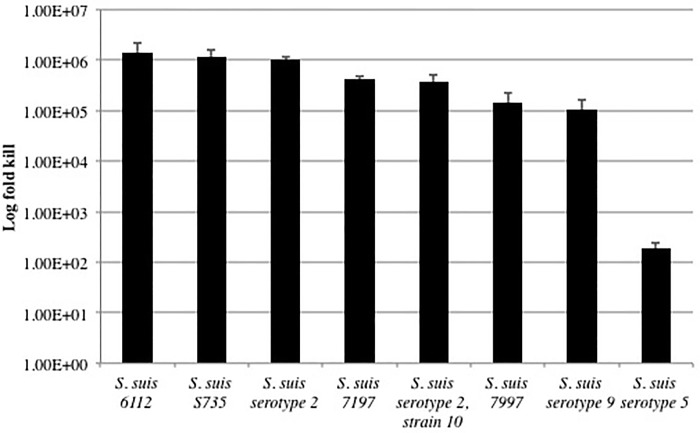
PlySs2 was bactericidal to nearly all strains of *S*. *suis*. Bacteria were grown to log-phase. After exposure to 64 μg/ml PlySs2 in buffer A for 60 min in 96-well plates, bacteria were serially diluted and plated to BHI agar for CFU enumeration. The CFU numbers of most *S*. *suis* strains dropped by 5 to 6 logs after PlySs2 treatment including the type strain S735 and the pathogenic strains 10 and 7997. Death (log fold kill) was calculated as -log[(CFUs in the test condition) ÷ (CFUs in the control condition)].

The lethal effect of PlySs2 was quantified for a select set of *S*. *suis* serotypes and strains. After 60 minutes of exposure to 64 μg/ml of PlySs2, all *S*. *suis* strains tested were reduced by 5–6 logs, except serotype 5 (~2-logs) (Fig 2). The relative lethal effect from one strain to another correlated with the lytic activity reported above. Strain 7197 was an outlier in that it was reduced by ~5.5 logs in the bactericidal assay, but was only reduced in the lytic assay to an OD_600_ ratio of ~0.5.

The MIC of PlySs2 against these serotypes also qualitatively correlated with the lytic and bactericidal assays. The PlySs2 MIC ranged from 32 to 512 μg/ml for all PlySs2-sensitive strains. As expected, serotype 12 was not inhibited at >1,024 μg/ml (Table 1).

**Table 1:** MIC of PlySs2 against *S*. *suis* strains^a^.

Species	Serotype	Strain	MIC (ug/ml)
*S*. *suis*	2	S735	32
*S*. *suis*	2		64
*S*. *suis*	7	7197	128
*S*. *suis*	9	7997	128
*S*. *suis*	9		128
*S*. *suis*	1	6112	256
*S*. *suis*	2	10	256
*S*. *suis*	5		512
*S*. *suis*	12		>1,024
*S*. *aureus*		MW2	16
*S*. *pyogenes*	M1	MGAS 5005	128

^*a*^ Bacteria were examined for growth at each concentration of PlySs2 from 0.5–1,024 μg/ml. The lowest concentration preventing growth is the PlySs2 MIC (column 4) for each strain (columns 2 and 3) of each species (column 1). Consistent with other tests, *S*. *suis* type strain S735 registered a low MIC while there was a higher MIC observed for *S*. *suis* strain 7997. The MIC of PlySs2 for *S*. *suis* serotype 12 was above the assay parameters. The previously published PlySs2 MICs for *S*. *aureus* and *S*. *pyogenes* are included for reference [25].

### Resistance to PlySs2

**Fig 3:**
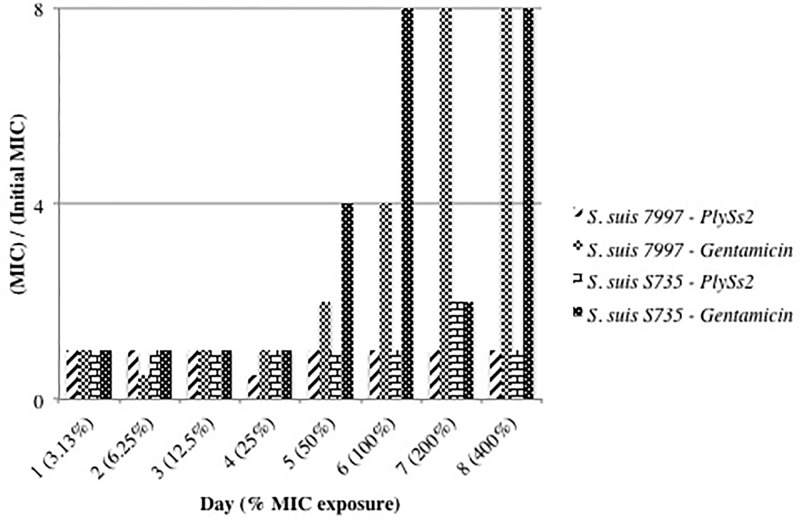
*S*. *suis* 7997 and S735 did not develop resistance to PlySs2 *in vitro*. *S*. *suis* strain S735 or *S*. *suis* strain 7997 grew in media containing 1/32× (3.13%) to 4× (400%) the MIC of PlySs2 or gentamicin over 8 days. Comparing the MICs of PlySs2 after each day to the initial MIC of PlySs2 for each strain determined resistance. Neither developed resistance to PlySs2. Both *S*. *suis* strain S735 and *S*. *suis* strain 7997 developed resistance to the positive control, gentamicin. The fluctuation observed in this assay was +/- 1x MIC.

According to an established protocol [25, 27, 28], both *S*. *suis* serotypes 2 and 9 (strains S735 and 7997 respectively) were challenged with incrementally doubling concentrations of PlySs2 to determine if they would develop resistance. Neither strain developed resistance–defined as exhibiting a >4-fold increase in MIC from the original PlySs2 MIC for each strain (Fig 3). Using the antibiotic gentamicin in the same protocol led to both strains S735 and 7997 developing resistance (Fig 3).

### *S*. *suis* intranasal mucosa decolonization

**Fig 4:**
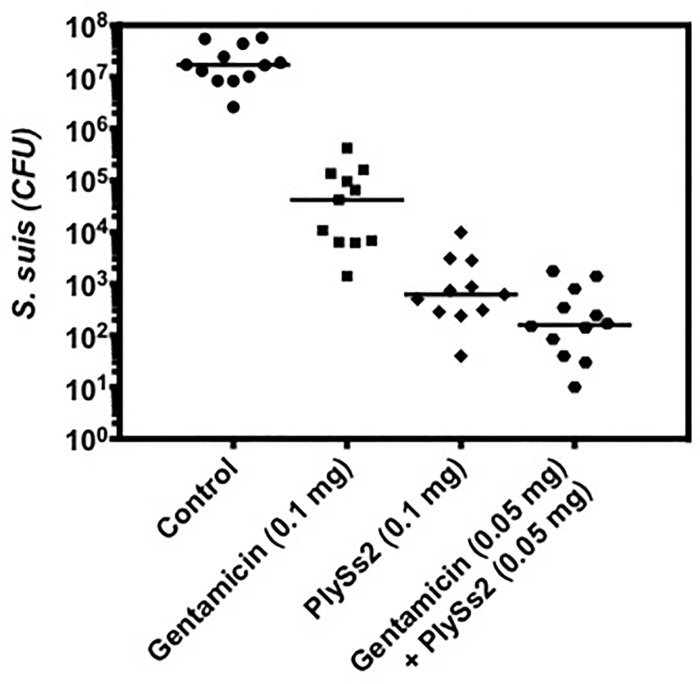
PlySs2 and gentamicin may act additively to reduce *S*. *suis in vivo*. PlySs2 removed *S*. *suis* from the murine intranasal mucosa. CD-1^®^ mice were nasally colonized with the pathogenic *S*. *suis* strain 7997. Twenty-four hours after colonization, in each nostril, mice received 10 μl of either 50 mM PB, pH 7.4 (buffer C), 5 mg/ml PlySs2 in buffer C, 5 mg/ml gentamicin in buffer C, or a combination of 2.5 mg/ml PlySs2 and 2.5 mg/ml gentamicin in buffer C. *S*. *suis* CFU counts were calculated for the nasal passage of each mouse.

To determine if the *in vitro* activity of PlySs2 against *S*. *suis* predicts its ability to remove *S*. *suis* colonizing the nasal passages *in vivo*, mice were intranasally colonized with *S*. *suis* strain 7997, and subsequently treated intranasally with a single dose of PlySs2 or buffer. The number of CFUs remaining in the nasal mucosa was determined by plating serial dilutions on blood agar. The results from multiple, separate experiments were combined and plotted (Fig 4). Relative to the buffer-treated control, the nasal mucosa of mice were decolonized of *S*. *suis* by >3 logs after gentamicin treatment, >4 logs after PlySs2 treatment, and >5 logs after treatment with gentamicin + PlySs2, each at half their dose (Fig 4).

## Discussion

In this study, the majority of examined *S*. *suis* strains were found to be very sensitive to the PlySs2 lysin as seen in lytic, bactericidal, and MIC assays. Neither the *S*. *suis* type strain S735 nor the pathogenic *S*. *suis* type 9 strain 7997 developed resistance to PlySs2 when tested *in vitro*. Furthermore, PlySs2 was found to be one of the most effective lysins used to decolonize the murine intranasal mucosa of pathogenic bacteria [15, 16, 18, 19, 30, 31]. When tested in combination with gentamycin against *S*. *suis in vivo*, it was more effective than each compound alone. A critical strength of PlySs2 is its specificity to a subset of Gram-positive bacterial pathogens, including *S*. *suis*, *S*. *pyogenes* and *S*. *aureus* [25], enabling broader protection. Our results show that PlySs2 can kill *S*. *suis in vivo* several-fold more effectively than gentamicin, without killing as broadly as gentamicin. During treatment of an *S*. *suis* infection with antibiotics, both commensal and pathogenic bacteria would be affected, causing potential deleterious effects in the treated individuals. However, PlySs2 used alone to treat a *S*. *suis* infections would have minimal effect on the normal bacterial flora.

In a recent publication, Tang and colleagues identified a *S*. *suis* lysin they termed Ply30 isolated from *S*. *suis* phage (24). In their *in vitro* studies, this enzyme was effective in killing all serotypes of *S*. *suis* tested to a maximum of about 2-logs using 50 ug/ml for 1 h of exposure compared to PlySs2 at 64ug/ml killing ~6-logs in the same period. Despite this difference in killing capacity, their MIC results were similar to the strains we tested (in the range of 32ug/ml to 64ug/ml). The reason for this discrepancy is unclear. While the authors found that the Ply30 lysin was effective in a mouse model of *S*. *suis* bacteremia, they did not use the enzyme for *S*. *suis* decolonization, so a direct comparison cannot be made. However, PlySs2 was previously shown to be effective in a similar bacteremia model using *S*. *aureus* and *S*. *pyogenes* as the bacteremic organisms (25).

A pregnant sow carrying *S*. *suis* in her upper respiratory mucosa may transmit the pathogen to her piglets after birth through nasal and oral shedding [32, 33], resulting in ~5% fatal infections [34–38]. PlySs2 could be used to prophylactically remove or significantly reduce *S*. *suis* from the nasal and oral mucosa of the pregnant or nursing sow until her offspring are beyond the age of acute *S*. *suis* susceptibility.

The only tested serotype unaffected by PlySs2, was serotype 12 (Fig 1). The reason for this resistance is unknown at this time, but could be due to differences in the cell wall structure or composition that either prevents lysin access to the peptidoglycan, or modifications in the binding or catalytic substrates of the serotype 12 cell wall. Nevertheless, all other strains tested, including both pathogenic serotypes 2 and 9 were highly sensitive to PlySs2.

There was consistency among the lytic, bactericidal, and MIC assays; each of which returned qualitatively correlative results–i.e., the most sensitive strains displayed high susceptibility in each assay. The MIC of other *S*. *suis* lysins have not been published, but the PlySs2 MIC for *S*. *aureus* and *S*. *pyogenes* are similar to those of *S*. *suis* (Table 1, [25]). For many clinical strains of *S*. *suis*, the MICs of ampicillin, amoxicillin, ciprofloxacin, kanamycin, and rifampin is >640 μg/ml [23]. This MIC level is higher than the PlySs2 MIC against all but one of the *S*. *suis* strains tested (Table 1). However, on a molar basis, with the molecular weight of PlySs2 being 26,060 g/mol, PlySs2 is several-fold more effective than antibiotics, which are usually ≤500 g/mol (e.g., gentamicin is ~478 g/mol).

Colonization by *S*. *suis* could be reduced by PlySs2 alone or in combination with gentamicin. An important finding in this report was that a single dose of PlySs2 could reduce *S*. *suis* on the intranasal mucosa by >4 logs (Fig 4). This is significantly greater than the <3-log reduction after treatment with gentamicin. PlySs2 + gentamicin resulted in a >5-log decrease in intranasal carriage after a single dose, suggesting that such a combination can be used together for increased effectiveness and at reduced doses of gentamycin. This supports previous studies reporting beneficial interactions between lysins and antibiotics–the first being the *in vitro* synergy of Cpl-1 with gentamicin and penicillin [39]. *In vivo* synergy has been reported between ClyS and oxacillin [18], and between PlySs2 with daptomycin, vancomycin, or oxacillin [40]. Other lysins have been shown to decolonize staphylococci, streptococci, or pneumococci in oral and nasal animal models [15, 16, 18, 19, 30, 31], but these lysin decolonization models did not include antibiotic combinations, and none were tested against *S*. *suis*. Our results indicate that lysins could be used in combination with antibiotics for mucosal decolonization, capitalizing on the strengths of both lysins (rapid, specific killing) and antibiotics (longer half life).

Using the same conditions that led to gentamicin resistance, the serotype 2 and 9 pathogenic *S*. *suis* were unable to establish resistance to PlySs2. This result is consistent with results of other lysins, such as ClyS and PlyG [28, 31], and other PlySs2-sensitive species such as MRSA and *S*. *pyogenes* [25]. To establish resistance, bacteria must inactivate, or remove the antimicrobial or alter the antimicrobial target. No molecule has yet been described to extracellularly inactivate any reported lysin. Because PlySs2 lyses disparate bacterial species with either diverse peptidoglycan cross-bridge structures, or no cross-bridge at all [41], the PlySs2 cleavage site in the bacterial cell wall is unlikely to be the cross-bridge, but the more common peptidoglycan structure. Since lysins have evolved to target essential cell wall structures [14], it may be difficult for resistance to rapidly occur.

In summary, we have presented a novel approach for the prevention of *S*. *suis* infection and/or colonization, with a phage lysin active against all but one tested strain of *S*. *suis*. While these experiments need to be repeated in pigs, it presents promising data for the use of lysins to reduce *S*. *suis* infections. It is possible that pregnant sows treated prophylactically (orally and intranasally) with PlySs2 alone or in combination with antibiotics prior to delivery, could help control this disease on the farm; newborn piglets could also be likewise treated for added control. Since we found that neither *S*. *suis* serotypes 2 and 9 strains tested developed resistance to PlySs2 *in vitro*, PlySs2 could be developed as a vital addition to the current approaches controlling *S*. *suis* spread in pigs, and zoonotic transmission.

## Supporting Information

S1 Table Strains used in this study. ^*a*^ 1, The Rockefeller University Collection; 2, Jaap A. Wagenaar, Utrecht University, Utrecht, Netherlands.
(DOCX)

S1 Fig PlySs2 displayed activity against almost all strains of *S*. *suis*. Bacteria in logarithmic growth were exposed to 32 μg/ml PlySs2 for 60 minutes in PB (for 30-minute readings, see Fig 1). The activity was measured by OD_600_ reduction. To normalize and combine values from multiple tests, the final OD_600_ of the treated samples was divided by the final OD_600_ of the untreated samples. An OD_600_ ratio of 1.0 indicates no lysis, while an OD_600_ ratio of ~0.02 indicates complete lysis.
(TIFF)

S2 Fig *S*. *suis* strain S735 is sensitive to concentrations as low as 0.5 μg/ml PlySs2. *S*. *suis* strain S735 in logarithmic growth was exposed to various concentrations of PlySs2 ranging from 0.25 μg/ml– 128 μg/ml for 60 minutes in PB. Readings at OD_600_ were taken every minute.
(TIFF)

S3 Fig *S*. *suis* strain 7997 is sensitive to concentrations as low as 2.0 μg/ml PlySs2. *S*. *suis* strain 7997 in logarithmic growth was exposed to various concentrations of PlySs2 ranging from 0.25 μg/ml– 128 μg/ml for 60 minutes in PB. Readings at OD_600_ were taken every minute.
(TIFF)
