# Supplementary material for: The Phage Lysin PlySs2 Decolonizes Streptococcus suis from Murine Intranasal Mucosa
Source: PLoS One. 2017 Jan 3;12(1):e0169180. doi: 10.1371/journal.pone.0169180 (PMC5207509; doi:10.1371/journal.pone.0169180)
Supplement: S1 Table — a 1, The Rockefeller University Collection; 2, Jaap A. Wagenaar, Utrecht University, Utrecht, Netherlands. (DOCX) [file pone.0169180.s001.docx]

**Table S1 Strains used in this study**

| **Species** | **Serotypes** | **Strain** | **ATCC** | **Source^a^** |
| --- | --- | --- | --- | --- |
|  |  |  |  |  |
| *Escherichia coli* |  | TOP10 |  | 1 |
| *Streptococcus suis* | 1 |  |  | 2 |
| *Streptococcus suis* | 1 | 6112 |  | 2 |
| *Streptococcus suis* | 1 | 6388 |  | 2 |
| *Streptococcus suis* | 2 |  |  | 2 |
| *Streptococcus suis* | 2 | 10 |  | 2 |
| *Streptococcus suis* | 2 | S375 |  | 2 |
| *Streptococcus suis* | 3 |  |  | 2 |
| *Streptococcus suis* | 4 |  |  | 2 |
| *Streptococcus suis* | 5 |  |  | 2 |
| *Streptococcus suis* | 6 |  |  | 2 |
| *Streptococcus suis* | 7 |  |  | 2 |
| *Streptococcus suis* | 7 | 7197 |  | 2 |
| *Streptococcus suis* | 7 | 7711 |  | 2 |
| *Streptococcus suis* | 8 |  |  | 2 |
| *Streptococcus suis* | 9 |  |  | 2 |
| *Streptococcus suis* | 9 | 7997 |  | 2 |
| *Streptococcus suis* | 9 | 8067 |  | 2 |
| *Streptococcus suis* | 10 |  |  | 2 |
| *Streptococcus suis* | 11 |  |  | 2 |
| *Streptococcus suis* | 12 |  |  | 2 |
| *Streptococcus suis* | 13 |  |  | 2 |
| *Streptococcus suis* | 14 |  |  | 2 |
