# Supplementary figures and images for: The Phage Lysin PlySs2 Decolonizes Streptococcus suis from Murine Intranasal Mucosa
Source: PLoS One. 2017 Jan 3;12(1):e0169180. doi: 10.1371/journal.pone.0169180 (PMC5207509; doi:10.1371/journal.pone.0169180)

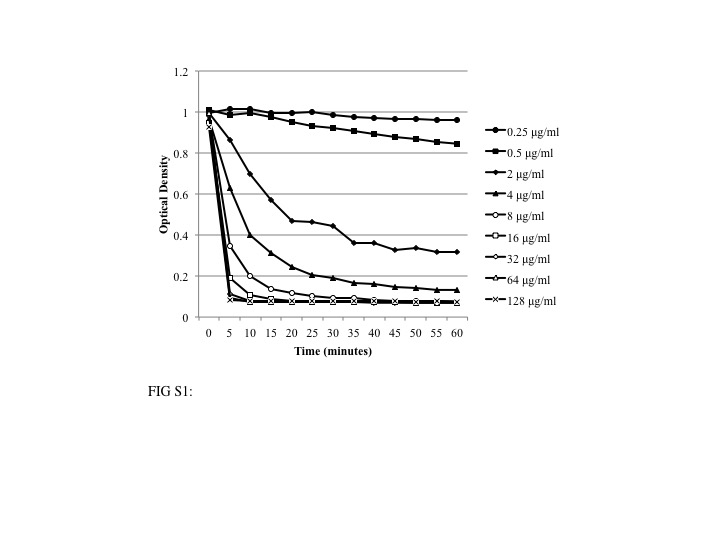

Supplement: S1 Fig — Bacteria in logarithmic growth were exposed to 32 μg/ml PlySs2 for 60 minutes in PB (for 30-minute readings, see Fig 1). The activity was measured by OD600 reduction. To normalize and combine values from multiple tests, the final OD600 of the treated samples was divided by the final OD600 of the untreated samples. An OD600 ratio of 1.0 indicates no lysis, while an OD600 ratio of ~0.02 indicates complete lysis. (TIFF) [file pone.0169180.s002.tiff]

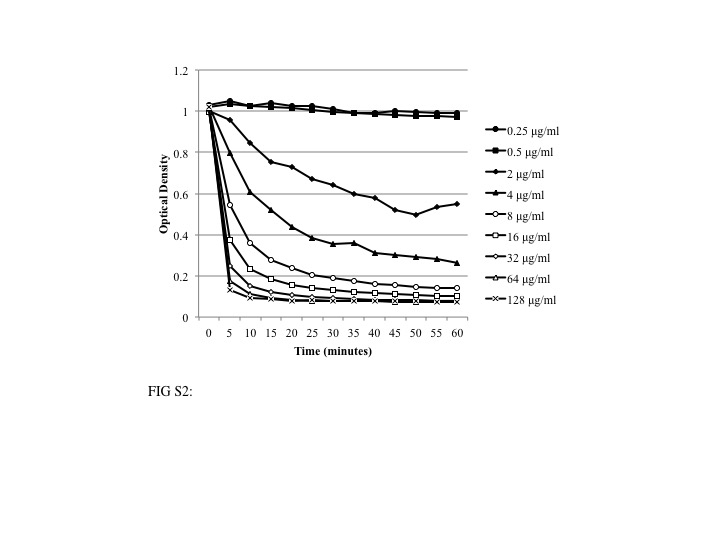

Supplement: S2 Fig — S. suis strain S735 in logarithmic growth was exposed to various concentrations of PlySs2 ranging from 0.25 μg/ml– 128 μg/ml for 60 minutes in PB. Readings at OD600 were taken every minute. (TIFF) [file pone.0169180.s003.tiff]

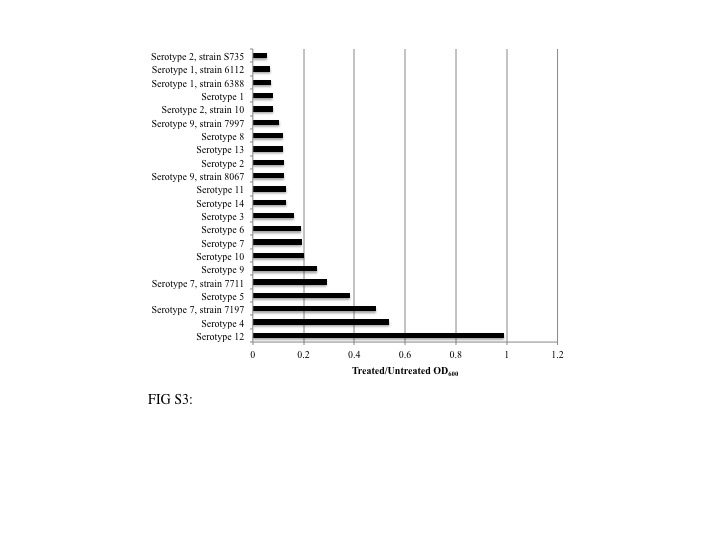

Supplement: S3 Fig — S. suis strain 7997 in logarithmic growth was exposed to various concentrations of PlySs2 ranging from 0.25 μg/ml– 128 μg/ml for 60 minutes in PB. Readings at OD600 were taken every minute. (TIFF) [file pone.0169180.s004.tiff]
